# Impact of Air Exposure on Vasotocinergic and Isotocinergic Systems in Gilthead Sea Bream (*Sparus aurata*): New Insights on Fish Stress Response

**DOI:** 10.3389/fphys.2018.00096

**Published:** 2018-02-13

**Authors:** Arleta K. Skrzynska, Elisabetta Maiorano, Marco Bastaroli, Fatemeh Naderi, Jesús M. Míguez, Gonzalo Martínez-Rodríguez, Juan M. Mancera, Juan A. Martos-Sitcha

**Affiliations:** ^1^Department of Biology, Faculty of Marine and Environmental Sciences, University of Cádiz, Cádiz, Spain; ^2^Laboratorio de Fisiología animal, Departamento de Biología Funcional y CC. de la Salud, Facultad de Biología, Universidade de Vigo, Pontevedra, Spain; ^3^Department of Marine Biology and Aquacuture, Instituto de Ciencias Marinas de Andalucía, Consejo Superior de Investigaciones Científicas, Cádiz, Spain; ^4^Nutrigenomics and Fish Growth Endocrinology Group, Institute of Aquaculture Torre de la Sal, Consejo Superior de Investigaciones Científicas, Castellón, Spain

**Keywords:** acute stress, arginine vasotocin, isotocin, metabolism, *Sparus aurata*

## Abstract

The hypothalamus-pituitary-interrenal (HPI) and hypothalamus-sympathetic-chromaffin cell (HSC) axes are involved in the regulation of the stress response in teleost. In this regard, the activation of a complex network of endocrine players is needed, including corticotrophin-releasing hormone (Crh), Crh binding protein (Crhbp), proopiomelanocortin (Pomc), thyrotropin-releasing hormone (Trh), arginine vasotocin (Avt), and isotocin (It) to finally produce pleiotropic functions. We aimed to investigate, using the gilthead sea bream (*Sparus aurata*) as a biological model, the transcriptomic response of different endocrine factors (*crh, crhbp, pomc*s, *trh*), neuropeptides (*avt* and *it*), and their specific receptors (*avtrv1a, avtrv2*, and *itr*) in four important target tissues (hypothalamus, pituitary, kidney and liver), after an acute stress situation. We also investigated several stress hormones (catecholamines and cortisol). The stress condition was induced by air exposure for 3 min, and hormonal, metabolic and transcriptomic parameters were analyzed in a time course response (15 and 30 min, and 1, 2, 4, and 8 h post-stress) in a total of 64 fish (*n* = 8 fish per experimental group; *p* = 0.05; statistical power = 95%). Our results showed that plasma noradrenaline, adrenaline and cortisol values increased few minutes after stress exposure. At hypothalamic and hypophyseal levels, acute stress affected mRNA expression of all measured precursors and hormonal factors, as well as their receptors (*avtr*s and *itr*), showing the activation, at central level, of HPI, HSC, and Avt/It axes in the acute stress response. In addition, stress response also affected mRNA levels of *avtr*s and *itr* in the head kidney, as well as the steroidogenic acute regulatory protein (*star*) and tyrosine hydroxylase (*th*) expression, suggesting their participation in the HPI and HSC axes activation. Moreover, the pattern of changes in hepatic *avtr*s and *itr* gene expression also highlights an important role of vasotocinergic and isotocinergic pathways in liver metabolic organization after acute stress events. Our results demonstrate, both at transcriptional and circulating levels of several hormones, the existence of a complex activation of different endocrine pathways in *S. aurata* related to the stress pathways, where vasotocinergic and isotocinergic systems can also be considered key players of the acute stress response orchestration.

## Introduction

The global increase of worldwide aquaculture is associated with growing concerns about fish welfare and improving a better understanding in physiological behavior of neuroendocrine factors and neurotransmitters. In aquaculture, fish are often exposed to several stress conditions due to farming practices (high densities, transport or handling, among others) or changes in abiotic environmental factors (temperature, salinity, biochemical water quality, etc.). During a stressful situation, an adaptive compensatory specific response, mediated by the so called “general adaptation syndrome,” with two different endocrine responses, occurred. This includes (i) an initial adrenergic response throughout the hypothalamus-sympathetic-chromaffin cell (HSC) axis, which rapidly increases the concentration of plasma catecholamines (adrenaline and noradrenaline); and (ii) a subsequent enhancement in plasma cortisol levels due to activation of the hypothalamus-pituitary-interrenal (HPI) axis (Wendelaar Bonga, 1997; Schreck and Tort, 2016).

In spite of the role of catecholamines and cortisol in the stress response of teleosts, the stress axis orchestration requires the dominant positive feed-forward signals of corticotropin-releasing hormone (Crh), together with others hypothalamic and hypophysiotropic factors (Bernier et al., 2009; Cerdá-Reverter and Canosa, 2009; Gorissen and Flik, 2016). In fact, arginine vasotocin (Avt) and isotocin (It) hormones have been reported to be important players in this endocrine cascade, and they have been pointed out as good indicators of fish welfare (Kulczykowska et al., 2009; Skrzynska et al., 2017; Mancera et al., 2018). Avt and It, which are known as homologs of arginine vasopressin (AVP) and oxytocin (OXY) in mammals (Acher, 1993), are both neuromodulators in central nervous system (Goodson and Bass, 2000). The synthesis of these neuropeptides starts in magnocellular and parvocelular neurons in the hypothalamic *nucleus preopticus* and *nucleus lateralis tuberalis*, from where they can be transported to the neurohypophysis and released into the bloodstream. In addition, due to the presence and distribution of their specific receptors in a large number of target tissues, a pleiotropic action of these hormones has been suggested. Therefore, they are also involved in osmoregulation, brain neurotransmission and pituitary endocrine activity, feeding regulation, or metabolism (Warne et al., 2002; Balment et al., 2006; Kulczykowska, 2007; Gesto et al., 2014; Banerjee et al., 2017).

Different endocrine and metabolic pathways have been previously studied in gilthead sea bream (*Sparus aurata*) under different chronic stress situations (Tort et al., 2011; Martos-Sitcha et al., 2016). The involvement of Avt and/or It, under different stocking densities, fasting conditions or salinity challenges, has been shown for this fish species (Sangiao-Alvarellos et al., 2006; Mancera et al., 2008; Martos-Sitcha et al., 2013, 2014a; Skrzynska et al., 2017, 2018), as well as in others teleosts (Fryer and Leung, 1982; Haruta et al., 1991; Gilchriest et al., 2000). In addition, the physiological role of several endocrine players has also been assessed in different fish species under acute stressors, including air exposure, handling, or hypoxia (Arends et al., 1999; López-Patiño et al., 2014; Vera et al., 2014; Martos-Sitcha et al., 2018). In fact, air exposure is a common acute stressor in aquaculture, since handling procedures during animal husbandry and production cycle require exposing fish to air environments with a subsequent physiological disturbance. Nonetheless, to our knowledge, only scarce information on the role of vasotocinergic and isotocinergic systems, after acute stress situations, has been reported in teleost species, although the short-term involvement of Avt as a potent inhibitor of appetite in fish has been suggested to be closely related to the activation of the HPI axis (Gesto et al., 2014). The aim of this study was to analyze, using *S. aurata* as a model species due to its importance in Spanish and European aquaculture, the physiological response after an acute stress situation caused by air exposure during 3 min. Thus, we investigated the putative role of different components of the vasotocinergic and isotocinergic systems (pro-peptide mRNA levels and the gene expression of their specific receptors in different target tissues) as biomarkers of the acute stress response. All of this was integrated in combination with different players from other endocrine and metabolic systems, and will allow to extent our knowledge on the physiological and endocrinological orchestration produced by a common acute stressor in aquaculture.

## Materials and methods

### Animals and experimental conditions

Gilthead sea bream (*S. aurata*) juveniles (*n* = 64, 108.83 ± 1.48 g body mass) were provided by *Servicios Centrales de Investigación en Cultivos Marinos* (SCI-CM, CASEM, University of Cádiz, Puerto Real, Cádiz, Spain; Operational Code REGA ES11028000312). During the experiment, fish were maintained in the SCI-CM under natural photoperiod (February-March) for our latitude (36° 31′ 44″ N) and constant temperature (18–19°C). Animals were randomly distributed into sixteen 90-L tanks (*n* = 4; 4.8 kg m^−3^) and acclimated to laboratory conditions 15 days before experiment was initiated, showing normal feeding and behavioral patterns during this period. All procedures were approved by the Ethics and Animal Welfare Committee of the University of Cádiz and carried out according to the National (RD53/2013) and the current EU legislation (2010/63/EU) on the handling of experimental fish.

### Experimental design and sampling

After the acclimation, fish from 12 out of 16 tanks were captured all together and moved to a rigid mesh where they were air exposed for 3 min. After this, fish were returned to their respective tanks. Sampling started at 9:00 a.m. (time 0 h) and it was performed in the following experimental times: 15 and 30 min, and 1, 2, 4, and 8 h after air exposure, in duplicate. Previous studies demonstrated that this protocol is effective to activate stress system in *S. aurata*, as well as in other teleost species (Arends et al., 1999; Costas et al., 2011). In addition, the remaining four tanks constituted the control group without stress, which were sampled at 0 and 8 h after the start of the experiment. For each experimental and control time, 4 fish per tank (*n* = 8 fish per group) were sampled. The number of fish analyzed was established based on previous experiences to ensure that any additional stress factor, mainly related to higher stocking densities, could influence the parameters analyzed in our experimental model, as well as to guarantee a sufficient number of replicates but following the 3 r's in the ethical use of animal experimentation. The control group sampled at 8 h served to identify possible circadian rhythms at the levels of metabolites and gene expression patterns that could mask the results (Vera et al., 2014). Sampled fish were anesthetized with a lethal dose of 2-phenoxyethanol (1 mL L^−1^ in seawater) (Sigma, Cat. # P-1126). The anesthesia process in all procedures was completed in less than 1 min. Blood was collected from the caudal peduncle with ammonium-heparinized syringes (Sigma, H-6279, 25,000 units in 3 mL of saline 0.9% NaCl), and fish were subsequently killed by spinal sectioning. Plasma, obtained after the whole blood was centrifuged, was stored in several aliquots at −80°C until analysis. A portion of liver was removed, immediately frozen in liquid nitrogen, and finally stored at −80°C for metabolite analyses. In addition, a representative biopsy of liver and head kidney tissues, as well as whole pituitaries and both hypothalamic lobes were placed in Eppendorf tubes containing 500 % (v/w) of RNA*later* (Ambion®, Applied BioSystems). These samples were kept for 24 h at 4°C and then stored at −20°C until total RNA isolation was performed.

### Analytical methods

#### Hepatic and plasma metabolites

For the assessment of hepatic metabolite levels, livers were finely minced in an ice-cold petri dish, subsequently homogenized by mechanical disruption (Ultra-Turrax, T25 basic, IKA®-WERKE) with 7.5 vol. (w/v) of ice-cooled 0.6 N perchloric acid and neutralized after the addition of the same volume of 1 M KHCO_3_. Prior to centrifugation, an aliquot of each homogenate was taken for the analyses of triglycerides (TAG). The remaining homogenates were then centrifuged (30 min, 13,000 g, 4°C) and the supernatants were recovered, distributed in several aliquots, and stored at −80°C until used in metabolite assays.

The analyses of all plasmatic and hepatic metabolites were performed using specific commercial kits from Spinreact (Barcelona, Spain) (Glucose-HK Ref. 1001200; Lactate Ref. 1001330; TAG ref. 1001311) adapted to a 96-well microplate. Plasma protein concentrations were analyzed, after a 50-fold dilution, by the bicinchoninic acid method with a BCA protein kit (Pierce P.O., Rockford, USA) with bovine serum albumin serving as the standard. Liver glycogen levels were assessed using the method from Keppler and Decker (1974), in which the glucose obtained via glycogen breakdown (after subtracting the free glucose levels) is determined using the previously described commercial glucose kit. All the assays were run on an Automated Microplate Reader (PowerWave 340, BioTek Instrument Inc., Winooski, USA) controlled by KCjunior™ software. Standards and samples were measured in quadruplicate and duplicate, respectively.

#### Plasma catecholamines

Catecholamines levels in plasma were quantified by high performance liquid chromatography (HPLC) with electrochemical detection after plasma purification by deproteinization, and then followed by solid-phase extraction (SPE) (Gesto et al., 2013). Samples were processed as follows: a 100 μL plasma aliquot was initially treated with 25 μL 0.6 M perchloric acid (HClO_4_; Merck, Darmstadt, Germany) and centrifuged (14,000 × g, 4 min, 4°C) to obtain a supernatant, which was neutralized with 25 μL of 1 M KHCO_3_ (Merck). Following centrifugation (14,000 g, 1 min, 4°C) and dilution with 1 mL in ultrapure water, samples were used for the SPE procedure. SPE cartridges (1 mL−100 mg tubes, Discovery DSC-WCX, Supelco, Bellefonte, PA, USA) were conditioned with 1.5 mL of ultrapure water at a flow rate of 5 mL min^−1^. Samples were then applied to the conditioned columns at 1 mL min^−1^, after which the columns were washed twice with 1 mL of ultrapure water (5 mL min^−1^). Finally, the catecholamines were eluted with two 400-μL aliquots of 0.3 M HClO_4_ at 1 mL min^−1^. SPE recoveries for noradrenaline and adrenaline were above 96%. Aliquots (20 μL) of these eluates were directly injected into the HPLC system, which was equipped with a Jasco PU-2080 Plus pump, a 5 μm analytical column (Nucleosil C18, 150 mm length × 4.6 mm diameter; Phenomenex, Macclesfield, Cheshire, UK) and an ESA Coulochem II detector (Chelmsford, MA, USA). The detection system included an analytical cell (M5011) with oxidation potentials set at +40 mV (first electrode) and +400 mV (second electrode). The mobile phase was a mixture of 25 mM citric acid (Panreac, Barcelona, Spain), 25 mM Na_2_HPO_4_ (Merck), 25 μM Na_2_EDTA (Sigma, St Louis, MO, USA), 0.21 mM sodium 1-octanesulfonate (Fluka, Sigma) (Panreac); pH was adjusted to 3.4 with ortho-phosphoric acid and then methanol 1% (v/v) was added. After filtered (0.20 μm filter, Millipore, Bedford, MA, USA) and degassed by vacuum the mobile phase was pumped at an isocratic flow rate of 1.3 mL min^−1^ at room temperature. The sample peaks were quantified by comparing peak areas to those of appropriate external standards. Under these conditions, the detection limits per injection were 3 pg for noradrenaline and 5 pg for adrenaline, with a signal-to-noise ratio of 3. Chromatograms were acquired and integrated by using ChromNAV version 1.12 software (Jasco Corp., Tokyo, Japan).

#### Total RNA isolation

Total RNA was isolated from complete pituitaries using a NucleoSpin®RNA XS kit (Macherey-Nagel), whereas the NucleoSpin®RNA II kit (Macherey-Nagel) was used for total RNA extraction from hypothalamus, head kidney and liver. An on-column RNase-free DNase digestion was used for gDNA elimination by following the manufacturer's instructions. The amount of total RNA was spectrophotometrically measured at 260 nm with the BioPhotometer Plus (Eppendorf) and the quality determined using a 2100 Bioanalyzer with the RNA 6000 Nano Kit (Agilent Technologies). Only samples with an RNA integrity number (RIN) higher than 8.5, which is indicative of intact RNA, were used for real-time PCR (qPCR).

#### Quantification of mRNA expression levels

First, 50 ng of total RNA from the pituitary, or 500 ng of total RNA from the hypothalamus, head kidney and liver, were used for reverse transcription using a qSCRIPT™ cDNA Synthesis Kit (Quanta BioSciences) in a final volume of 20 μL. The qPCR was performed with a fluorescent quantitative detection system (Eppendorf Mastercycler ep realplex^2^ S). Each reaction mixture, in a final volume of 10 μL, contained 0.5 μL of each specific forward and reverse primers, 5 μL of PerfeCTa SYBR® Green FastMix™ 2x (Quanta BioSciences) and 4 μL containing either 1 ng or 10 ng of cDNA from the pituitary or from the hypothalamus, head kidney and liver, respectively.

Primers for *crh, crhbp, trh, pomca, pomcb, avt, it, avtrv1, avtrv2, itr*, from *S. aurata* (at the final concentration described in Table 1) were used as previously described by Martos-Sitcha et al. (2013, 2014a,b), Toni et al. (2015), and Ruiz-Jarabo et al. (2018), and designed from the nucleotide sequences available at NCBI website (*crh*, acc. no.: **KC195964**; *crhbp*, acc. no.**: KC195965**; *trh*, ac. no.: **KC196277**; *pomca*, acc. no.: **HM584909**; *pomcb*, acc. no.: **HM584910**; *avt*, acc. no.: **FR851924**; *it*, acc. no.: **FR851925**; *avtrv1a*, acc. no. : **KC195974**; *avtrv2*, acc. no.: **KC960488**; *itr*, acc. no.: **KC195973**). For *steroidogenic acute regulatory protein* (*star*, acc.no. **EF640987**; previously described by Castillo et al., 2008) and *tyrosine hydroxylase* (*th*, acc. no. **DQ072727**) genes, optimization of qPCR conditions on primer annealing temperature (50–60°C), primers concentration (100, 200, and 400 nM) and template concentration (six 1:10 dilution series from 10 ng to 100 fg of input RNA) was achieved. Moreover, two negative controls, with (i) RNA (10 ng/reaction) and (ii) sterile water, were performed to detect possible gDNA contamination or primer-dimer artifacts. Several curves with different template concentrations in serial dilutions for all primer pairs had amplification efficiencies and *r*^2^ of 0.97–1.03 and 0.990–0.998, respectively. The final PCR profile for all analyzed genes was as follows: 95°C, 10 min; [95°C, 20 s; 60°C, 30 s] × 40 cycles; melting curve [60–95°C, 20 min], 95°C, 15 s. The melting curve was used to ensure that a single product was amplified and to check the absence of primer-dimer artifacts. Results were normalized to β-actin (*actb*, acc. no. **X89920**), owing its low variability (<0.20 C_T_ in all tissues analyzed) under our experimental conditions. Relative gene quantification was performed using the ΔΔC_T_ method (Livak and Schmittgen, 2001).

**Table 1:** Specific primers used for the semi-quantitative qPCR expression analysis and sizes of the amplified products.

**Primers**	**Nucleotide sequence**	**Primer concentration (nM)**	**Amplicon size (bp)**
*crh*_F_	5′-ATGGAGAGGGGAAGGAGGT-3′	200	176
*crh*_R_	5′-ATCTTTGGCGGACTGGAAA-3′		
*crhbp*_F_	5′-GCAGCTTCTCCATCATCTACC-3′	200	147
*crhbp*_R_	5′-ACGTGTCGATACCGCTTCC-3′		
*trh*_F_	5′-GAAACGCTTTTGGGATAACTCC-3′	300	131
*trh*_R_	5′-CGGCGTGACTCTTGTTTATGTT-3′		
*pomca*_F_	5′-GGAGCCAGAAGAGAGAGCAGTGAT-3′	400	122
*pomca*_R_	5′-ATCGGGTCAGAAAACACTCA-3′		
*pomcb*_F_	5′-AGCTCGCCAGTGAGCTGT-3′	600	81
*pomcb*_R_	5′-CCTCCTGCATCACTTCCTG-3′		
*avt*_F_	5′-AGAGGCTGGGATCAGACAGTGC-3′	200	129
*avt*_R_	5′-TCCACACAGTGAGCTGTTTCCG-3′		
*it*_F_	5′-GGAGATGACCAAAGCAGCCA-3′	200	151
*it*_R_	5′-CAACCATGTGAACTACGACT-3′		
*avtrv1a*_F_	5′-GACAGCCGCAAGTGATCAAG-3′	400	203
*avtrv1a*_R_	5′-CCCGACCGCACACCCCCTGGCT-3′		
*avtrv*2_F_	5′-ATCACAGTCCTTGCATTGGTG-3′	600	120
*avtrv*2_R_	5′-GCACAGGTTGACCATGAACAC-3′		
*itr*_F_	5′-GGAGGATCGTTTTAAAGACATGG-3′	400	120
*itr*_R_	5′-TGTTGTCTCCCTGTCAGATTTTC-3′		
*star*_F_	5′-GCTGGATCCCAAAGACAATC-3′	300	175
*star*_R_	5′-CTTGCTCTCCTTCGACTGCT-3′		
*th*_F_	5′-CAGACTTGGATCAAGACCATCC-3′	300	136
*th*_R_	5′-CCGATCTCCTCCTCTGTGTACT-3′		
*actb*_F_	5′-TCTTCCAGCCATCCTTCCTCG-3′	200	108
*actb*_R_	5′-TGTTGGCATACAGGTCCTTACGG-3′		

### Statistical analysis

Sample size used (*n* = 8 fish per group) was confirmed to be enough for our experimental model adopting a *p* = 0.05 and a statistical power of 95%. Significant differences between groups were analyzed by one-way analysis of variance (one-way ANOVA), taking the experimental time from air exposure as the main factor (0 h, 15, and 30 min, 1, 2, 4, and 8 h). When significant differences were detected, the Tukey's test for comparison of the groups was used. Test for normality and homoscedasticity was run before the execution of the analysis of variance. In addition, a comparison of non-stressed groups at times 0 and 8 h, and between the groups with and without stress at time 8 h, as well as of replicate tanks for all of the parameters, a Student's *t*-test analysis was used. A significance level of *p* < 0.05 was adopted. All tests were performed using the GraphPad Prism® (v.5.0b) software for Macintosh.

## Results

No mortality, health disturbance or any alterations in fish behavior were observed in any experimental group.

### Plasma and liver metabolites

Metabolic responses at plasma and hepatic levels in *S. aurata* individuals under a 3 min air exposure challenge are shown in Table 2. Fish from the control group sampled at both 0 and 8 h did not show differences in the levels of all plasma metabolites assessed. In air-exposed fish, the levels of plasma glucose rapidly increased with time, being significantly higher 15 min after air exposure, reaching more than 2-fold increase at the end of the measured experimental time. Lactate values in stressed fish enhanced more than 4-fold within 15 min compared with unstressed fish, and then decreased to levels similar to control group. Later, a second and transient increase occurred 4 h after air exposure, which then decreased at the end of the experiment. Moreover, values of plasma proteins gradually decreased after air exposure, being statistically different only at 4 h after emersion. Triglyceride levels did not show significant differences along the experimental time. In addition, glucose levels were statistically enhanced in stressed fish along all the time compared to non-stressed individuals.

**Table 2:** Time course changes in plasma and hepatic metabolites in *S. aurata* exposed to air for 3 min. Values are represented as mean ± S.E.M. (*n* = 8 fish per group).

**Time**	**0 h**	**15 min**	**30 min**	**1 h**	**2 h**	**4 h**	**8 h**	**8 h without stress**
**Plasma metabolites**								
Glucose (mM)	4.16 ± 0.14^*a*^	6.18 ± 0.62^*b*^	6.53 ± 0.25^*bc*^	6.40 ± 0.49^*bc*^	6.99 ± 0.28^*bc*^	6.56 ± 0.44^*bc*^	8.49 ± 0.20^c^^*^	4.97 ± 0.15^*a*^
Lactate (mM)	7.34 ± 0.94^*a*^	34.14 ± 5.74^*b*^	12.99 ± 3.47^*ab*^	16.91 ± 3.42^*ab*^	5.73 ± 0.13^*a*^	29.31 ± 5.11^*b*^	6.78 ± 1.99^*a*^	5.13 ± 0.44^*a*^
Triglyceride (mM)	2.36 ± 0.25	2.55 ± 0.23	2.71 ± 0.36	2.94 ± 0.24	2.46 ± 0.22	2.59 ± 0.26	2.31 ± 0.15	2.13 ± 0.19
Proteins (mg/mL)	11.47 ± 0.47^*a*^	11.16 ± 0.41^*ab*^	10.63 ± 0.38^*ab*^	10.54 ± 0.67^*ab*^	10.57 ± 0.31^*ab*^	9.63 ± 0.43^*b*^	11.02 ± 0.56^ab^^*^	12.67 ± 0.12^*a*^
**Liver metabolites**								
Glucose (μmol/g ww)	30.86 ± 1.50^*a*^	37.95 ± 2.57^*b*^	26.79 ± 1.59^*a*^	31.91 ± 1.10^*ab*^	34.97 ± 1.92^*ab*^	37.06 ± 1.74^*ab*^	39.56 ± 3.50^ab^^*^	44.91 ± 2.44^*b*^
*G*lycogen (μmol/g ww)	154.62 ± 8.90^*a*^	138.56 ± 2.89^*ab*^	139.10 ± 5.73^*ab*^	143.72 ± 4.76^*ab*^	136.69 ± 3.55^*ab*^	134.46 ± 7.05^*ab*^	136.05 ± 4.51^ab^^*^	122.53 ± 6.61^*b*^
Lactate (μmol/g ww)	0.011 ± 0.001^*a*^	0.017 ± 0.001^*b*^	0.012 ± 0.001^*ab*^	0.013 ± 0.001^*ab*^	0.012 ± 0.001^*ab*^	0.012 ± 0.001^*ab*^	0.016 ± 0.001^b^^*^	0.009 ± 0.003^*a*^
Triglyceride (μmol/g ww)	0.09 ± 0.01	0.05 ± 0.02	0.08 ± 0.01	0.10 ± 0.01	0.10 ± 0.01	0.05 ± 0.02	0.08 ± 0.01	0.09 ± 0.011

Significant differences within each group for different experimental times are identified with different letters (p < 0.05, one-way ANOVA followed by Tukey's test); asterisks (^*^) indicate significant differences between groups at the same time (p < 0.05, Student's t-test).

At hepatic level, free glucose increased rapidly at 15 min after air exposure, but at 30 min values were similar to those of control group, increasing again until the end of the experimental time. Moreover, significant differences were found between both control groups (non-stressed fish), with higher values in the group sampled after 8 h compared to those of the group sampled at 0 h, as well as when compared with stressed fish at 8 h. In turn, hepatic glycogen levels clearly decreased from the first 15 min post-stress until the end of the experimental time, reaching similar values to those of the control group at time 0. Hepatic lactate presented a two-peak increase, during the first 15 min after the emersion and at the end of the trial, leading to a significant difference between both stressed and unstressed fish (control group) after 8 h of experiment. Triglyceride levels did not show significant differences, as observed in plasma.

### Endocrine response of HPI and HSC axes

**Figure 1:**
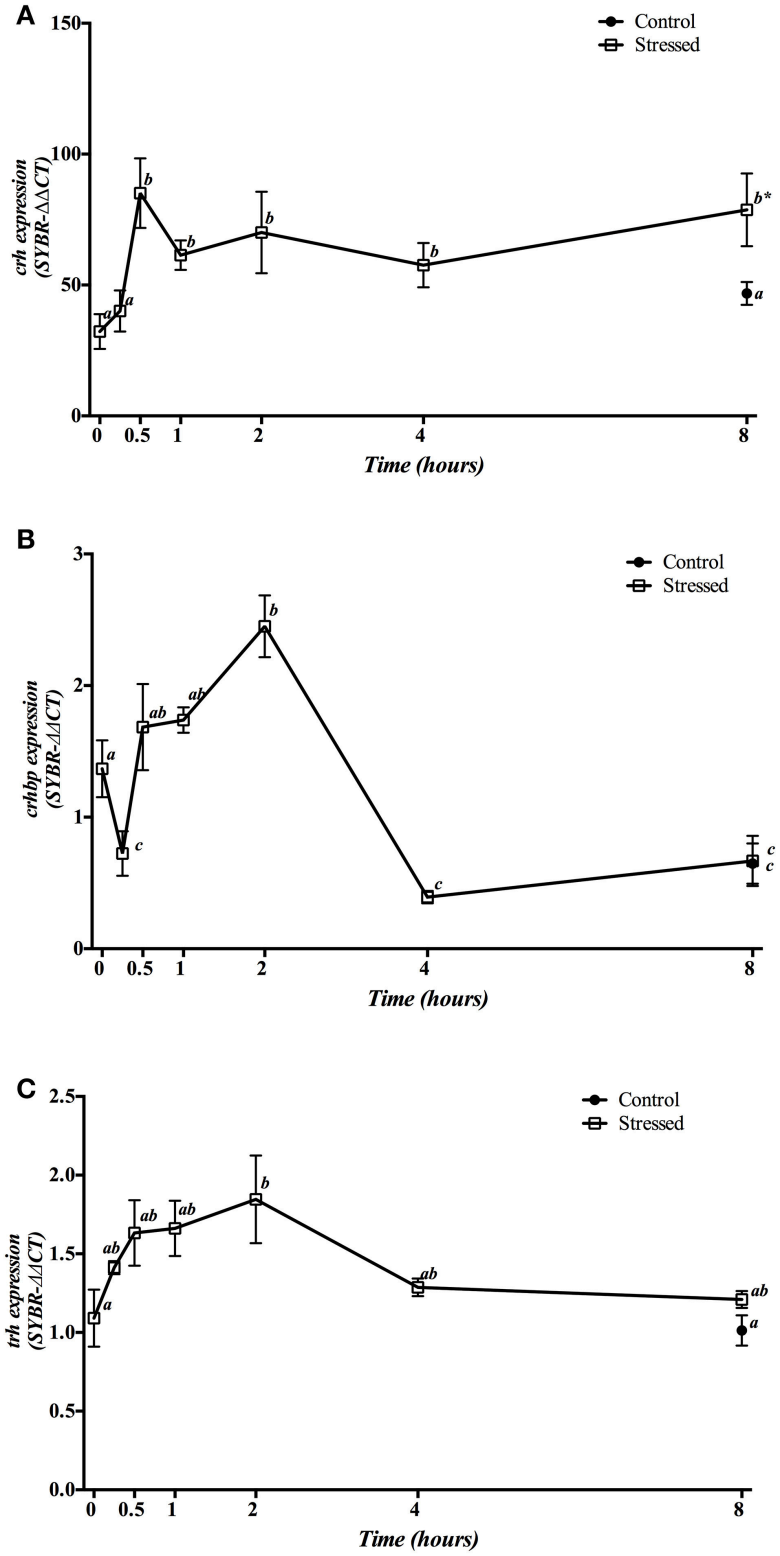
Time course changes in hypothalamic *crh* **(A)**, *crhbp* **(B)**, and *trh* **(C)** mRNA expression levels (relative to *actb*) in *S. aurata* exposed to air for 3 min. Values are represented as mean ± S.E.M. (*n* = 8 fish per group). Significant differences within each group for different experimental times are identified with different letters (*p* < 0.05, one-way ANOVA followed by Tukey's test); asterisks (^*^) indicate significant differences between groups at the same time (*p* < 0.05, Student's *t*-test).

Hypothalamic *crh* mRNA expression was significantly higher in fish under acute stress from 30 min after air exposure, which remained high until the end of experiment. No significant changes were observed in control group sampled at 8 h as compared with time 0 h group (Figure 1A). Meanwhile, *crhbp* gene expression levels showed a differential pattern along the time, with (i) a significant decrease during the first 15 min post-stress, (ii) a progressive increase which reached a maximum peak at 2 h, and (iii) a decrease to reach their minimum values after 4 h post-stress, then remaining low until the end of the experiment. Similarly, the control group sampled at 8 h significantly decreased its values compared with control fish at the beginning of the experiment, and did not differ from the stressed group after 8 h of air exposure (Figure 1B). Finally, *trh* gene expression enhanced significantly at 2 h post-stress, maintaining intermediate levels after that (Figure 1C).

**Figure 2:**
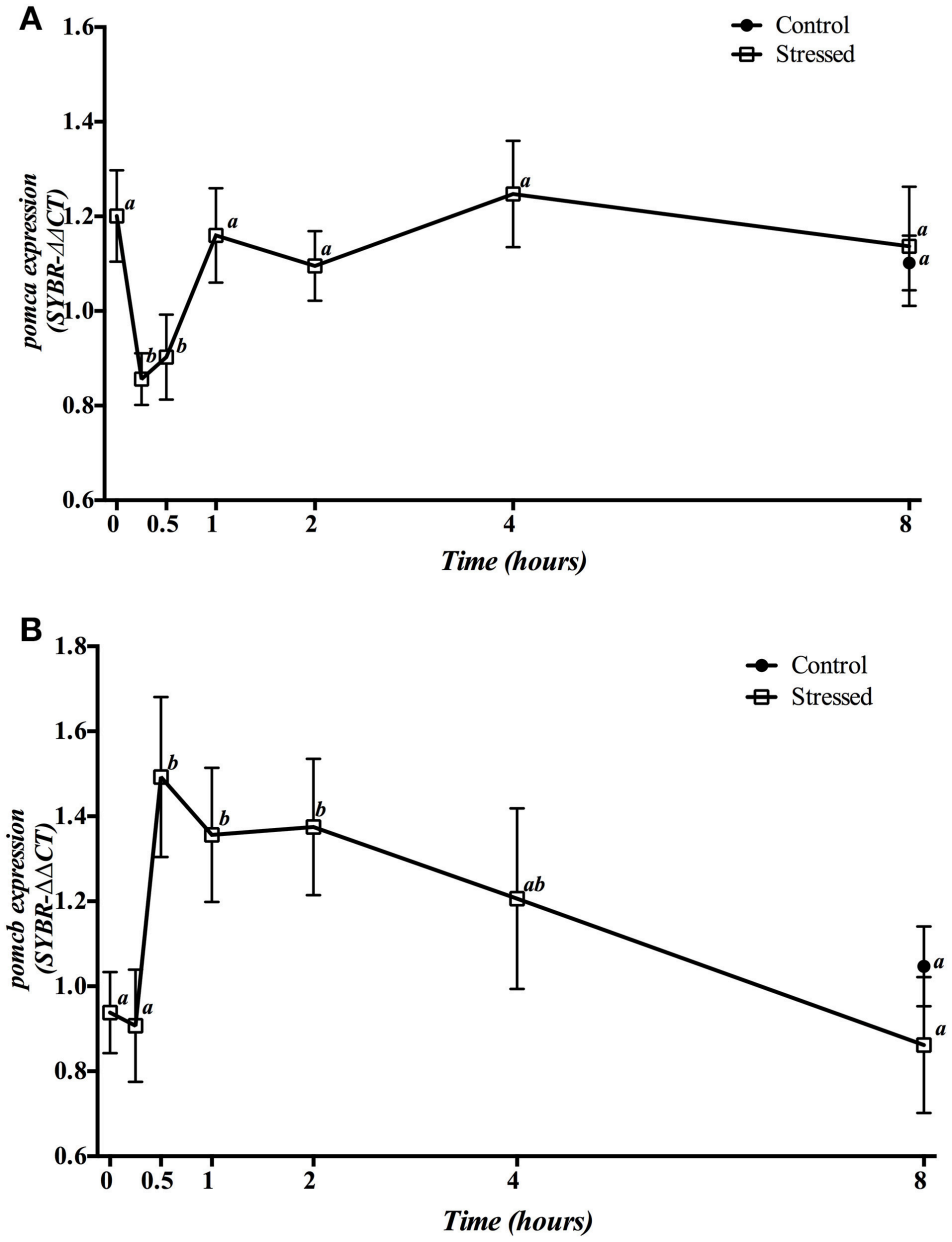
Time course changes in hypophyseal *pomca* **(A)** and *pomcb* **(B)** mRNA expression levels (relative to *actb*) in *S. aurata* exposed to air for 3 min. For further details, see the legend on Figure 1.

Gene expression of hypophyseal *pomca* and *pomcb* is presented in Figure 2. Three min of air exposure significantly decreased *pomca* expression, which remained low during the next 30 min, returning to baseline values from 1 h post-stress onwards (Figure 2A). Meanwhile, the expression of *pomcb* showed a significant increase from 30 min till 2 h post-stress, with a progressive decrease that reached control values at 8 h post-exposure (Figure 2B). Moreover, fish kept as control group and sampled at 8 h, showed no significant differences in the expression of both *pomca* and *pomcb* genes when compared to control group at time 0 as well as to the 8 h post-stress group.

**Figure 3:**
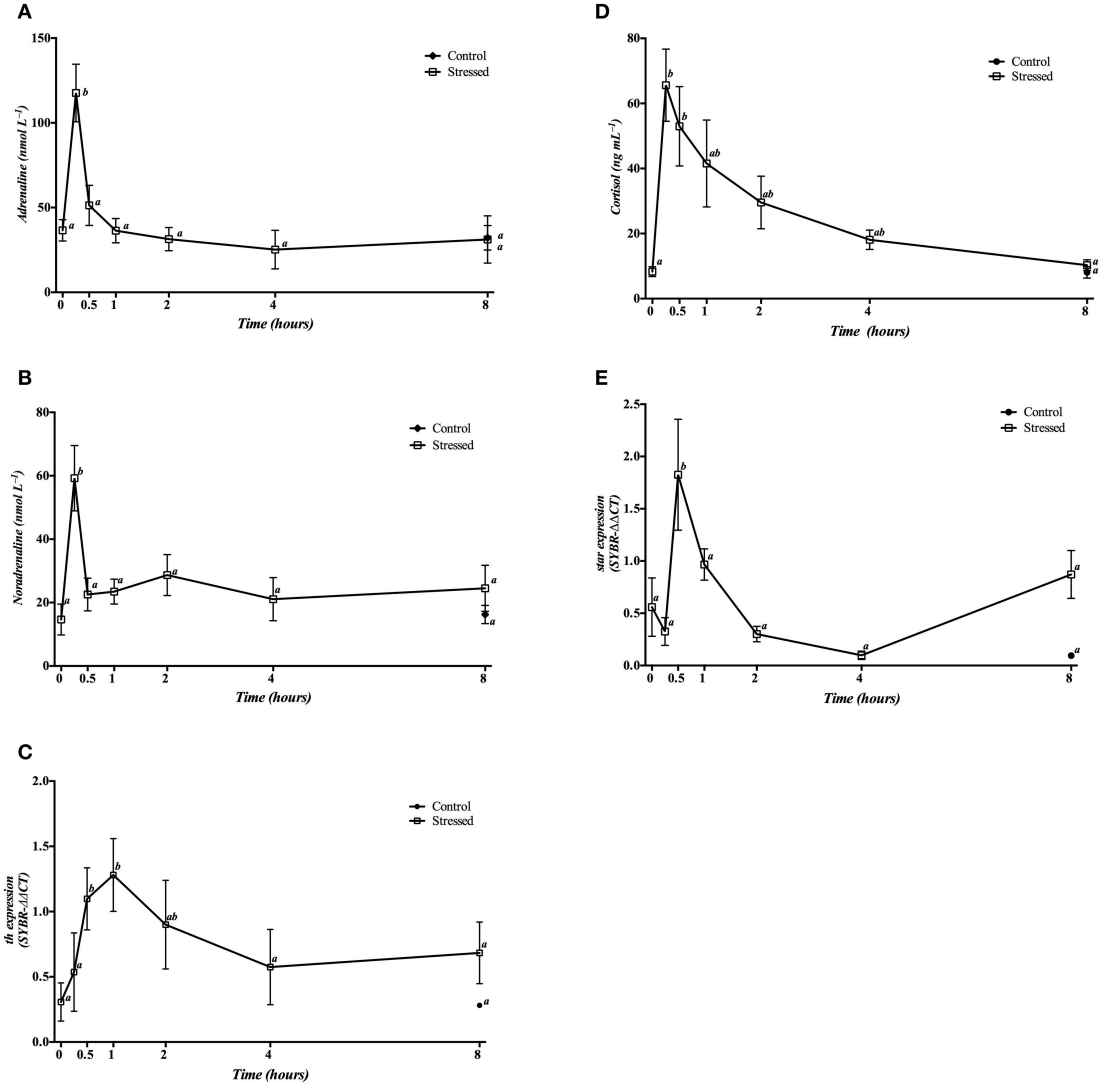
Time course changes in plasma adrenaline **(A)**, noradrenaline **(B)** and cortisol **(D)** levels, as well as in the head kidney expression levels of *th* **(C)** and *star* **(E)** genes (relative to *actb*) in *S. aurata* exposed to air for 3 min. For further details, see the legend on Figure 1.

Plasma catecholamines (adrenaline, and noradrenaline) showed a significant pronounced increase 15 min post-stress, returning to baseline values from 30 min post-stress onwards (Figures 3A,B). A significant increase was also observed in plasma cortisol levels at 15 and 30 min after air exposure, gradually decreasing over time until reaching then values similar to those found in the control groups (0 and 8 h) at the end of the trial (Figure 3D).

Expression of *th* mRNA was significantly increased 30 min and 1 h post-stress, eventually decreasing to control levels after 4 h (Figure 3C). Moreover, *star* gene expression only showed a significant increase 30 min post stress, decreasing to basal levels afterwards (Figure 3E).

No significant differences were observed in all of these parameters in control fish sampled at 8 h when compared with the time 0 group, or even with animals of the 8 h stress group.

### Changes in Avt and it pathways

**Figure 4:**
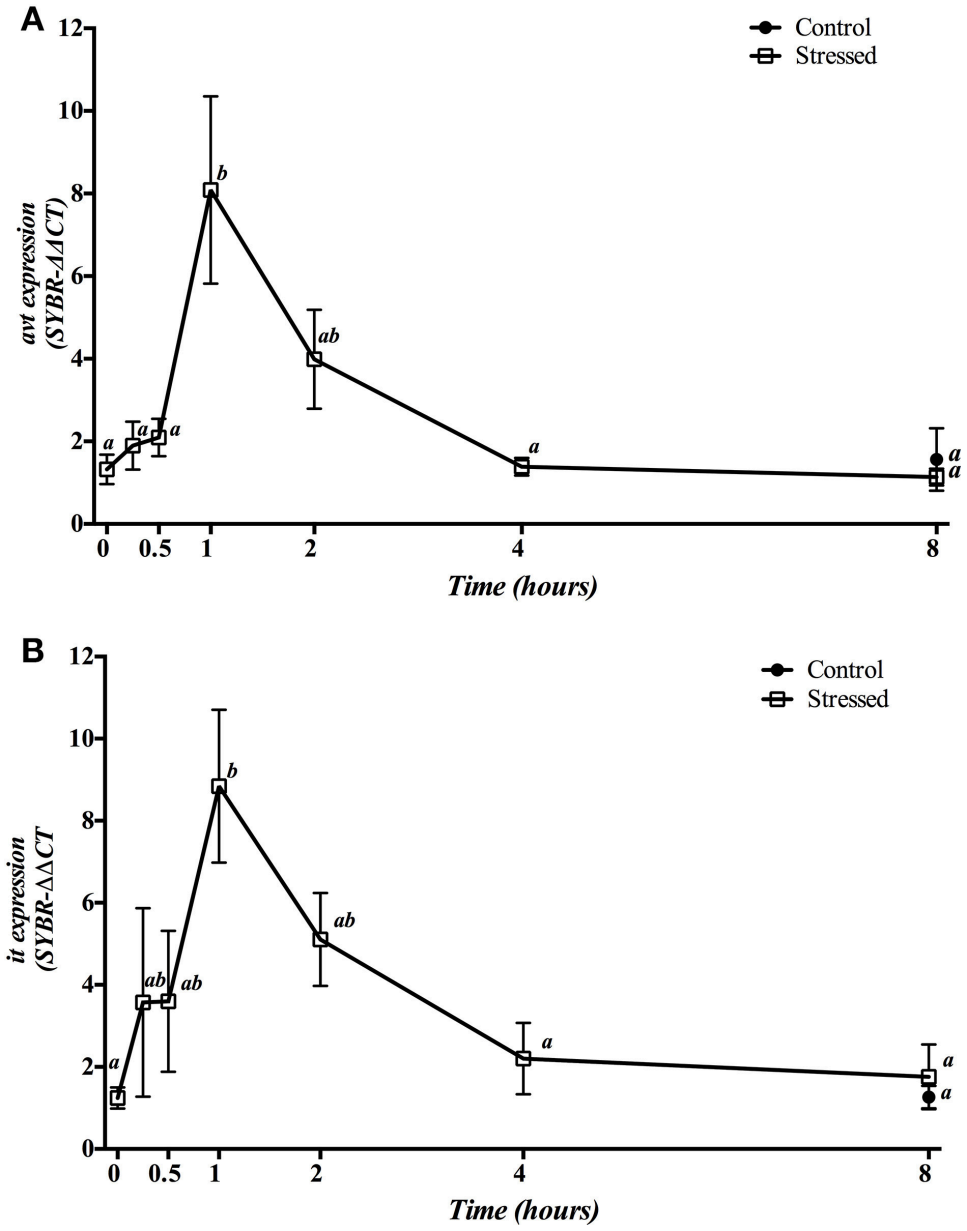
Time course changes in hypothalamic *avt* **(A)** and *it* **(B)** mRNA expression levels (relative to *actb*) in *S. aurata* exposed to air for 3 min. For further details, see the legend on Figure 1.

Three min of stress from air exposure significantly enhanced the hypothalamic gene expression of both *avt* (Figure 4A) and *it* (Figure 4B) 1 h later. Then, mRNA values gradually dropped to baseline values after 4 h. Meanwhile, fish kept as control group and sampled at 8 h showed no significant differences when compared to control group at time 0 h as well as to the 8 h post-stress group.

**Figure 5:**
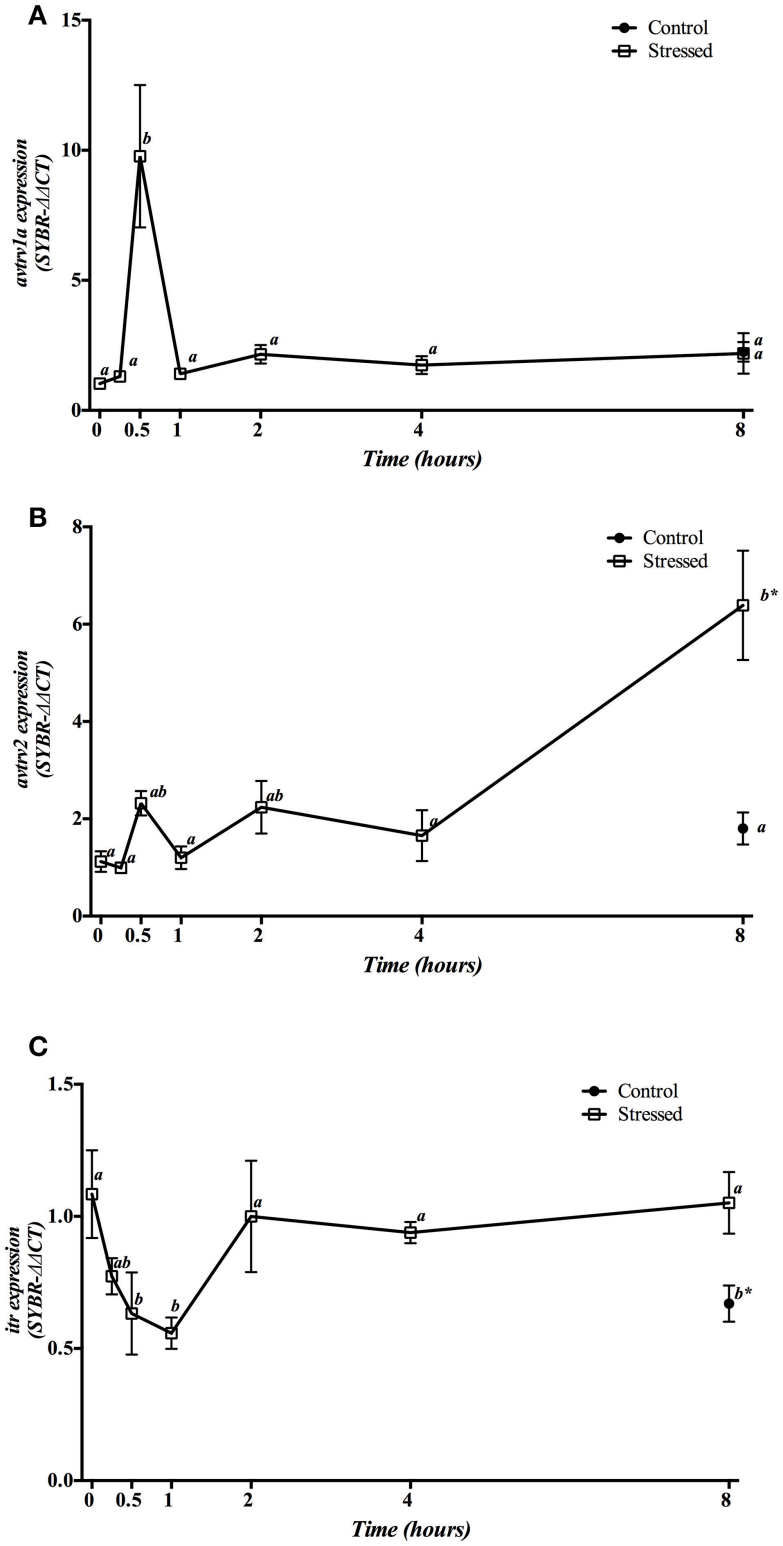
Time course changes in hypothalamic *avtrv1a* **(A)**, *avtrv2* **(B)**, and *itr* **(C)** mRNA expression levels (relative to *actb*) in *S. aurata* exposed to air for 3 min. For further details, see the legend on Figure 1.

Hypothalamic mRNA expression of Avt and It receptors showed a different pattern of change during the experimental period (Figure 5). The expression of *avtrv1a* significantly increased 30 min post-stress, being their basal levels rapidly restored after 1 h and remaining low until the end of the experiment (Figure 5A). However, *avtrv2* enhanced significantly at 8 h post-stress, being also significantly higher when compared with the 8 h control group (Figure 5B). Gene expression of both *avt receptor* types was not affected in the control groups at different sampling points (at times 0 h and 8 h) (Figures 5A,B). Moreover, *itr* mRNA expression in air-exposed fish was significantly decreased at 30 min and 1 h post-stress, recovering its basal values from 2 h onwards. In addition, fish maintained as control group and sampled at 8 h revealed a significant decrease when compared with both control fish at time 0 h as well as with stressed fish at 8 h post-induction (Figure 5C).

**Figure 6:**
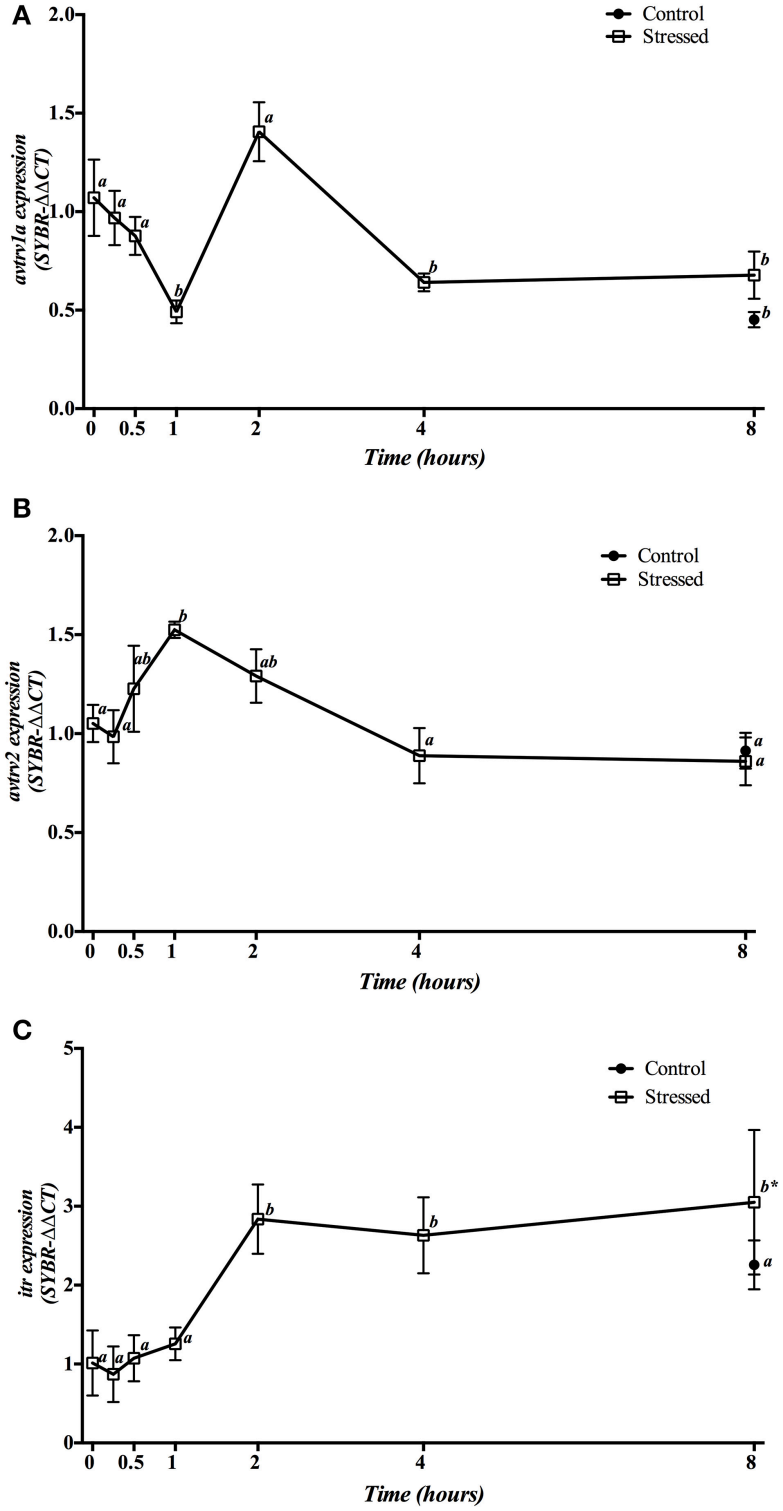
Time course changes in hypophyseal *avtrv1a* **(A)**, *avtrv2* **(B)**, and *itr* **(C)** mRNA expression levels (relative to *actb*) in *S. aurata* exposed to air for 3 min. For further details, see the legend on Figure 1.

Gene expression of hypophyseal *avt* and *it receptors* is presented in Figure 6. Expression of *avtrv1a* significantly decreased at 1 h post-stress, returning to control values at 2 h to finally reach a significant decrease from 4 h onwards. The control group sampled at 8 h did not show significant differences respect to the stressed groups at the same sampling point, but showed a significant decrease when compared to the control group at time 0 h (Figure 6A). Meanwhile, mRNA expression of *avtrv2* statistically increased 1 h after air exposition, decreasing then to reach the control values at the end of the experiment. The control group sampled at 8 h did not present significant differences compared with the time 0 h group nor with stressed fish after 8 h post-induction (Figure 6B). Finally, *itr* mRNA expression significantly increased in fish under acute stress from 2 h onwards, presenting significant differences with the 8 h control group, which did not show significant differences when compared with the time 0 h control group (Figure 6C).

**Figure 7:**
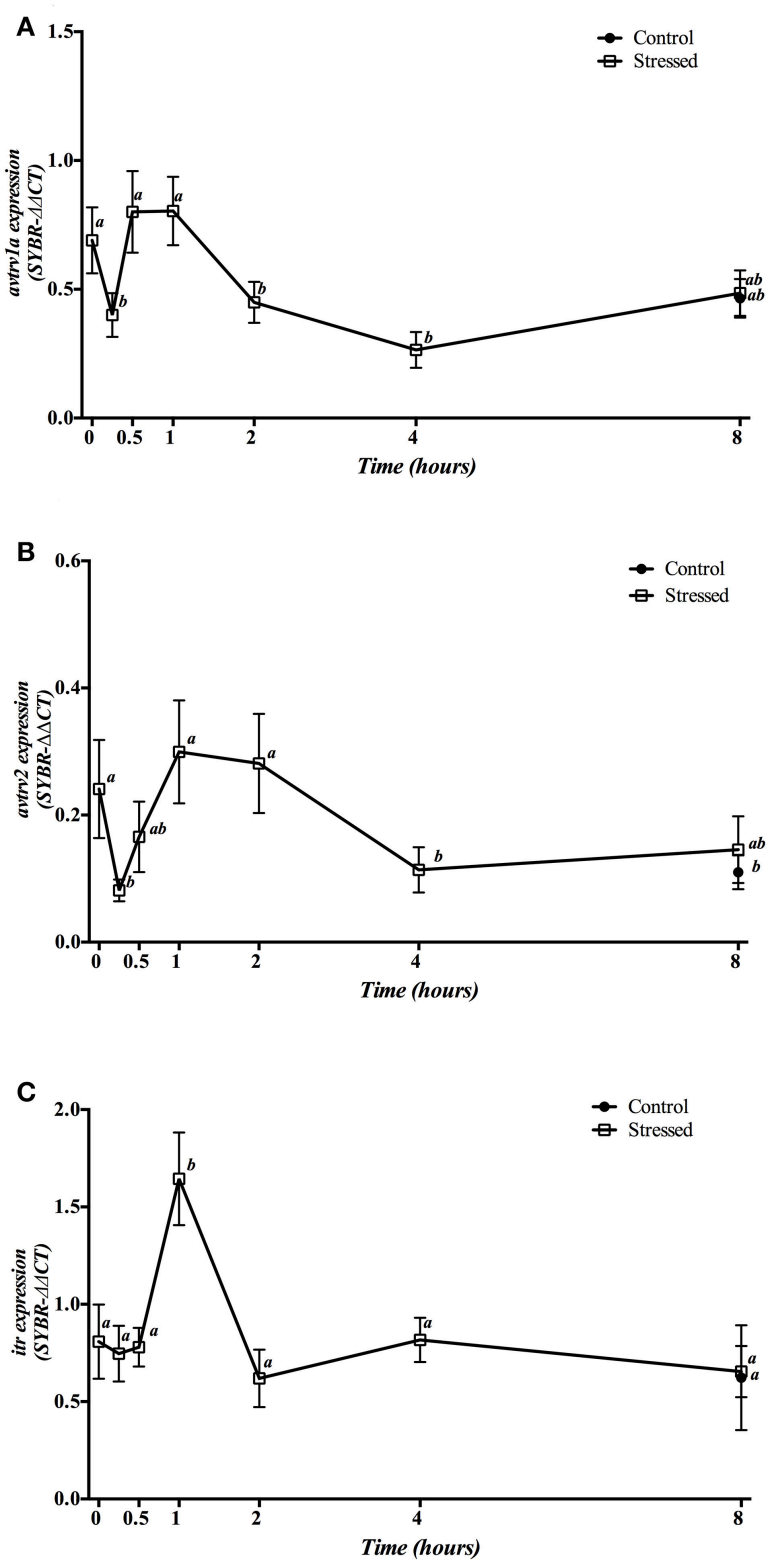
Time course changes in kidney *avtrv1a* **(A)**, *avtrv2* **(B)**, and *itr* **(C)** mRNA expression levels (relative to *actb*) in *S. aurata* exposed to air for 3 min. For further details, see the legend on Figure 1.

Gene expression of *avt* and *it receptors* in the head kidney is shown in Figure 7. mRNA expression of both *avtrv1a* and *avtrv2* showed a double response with a sharp decrease 15 min after stress, reaching its basal and control values between 30 min and 1 h post-stress, and decreasing again at 4 h post-stress (Figures 7A,B), whereas *itr* gene expression enhanced at 1 h post-stress (Figure 7C). The group sampled 8 h post-stress did not show significant differences in any of the receptors studied when compared with both control groups (times 0 and 8 h).

**Figure 8:**
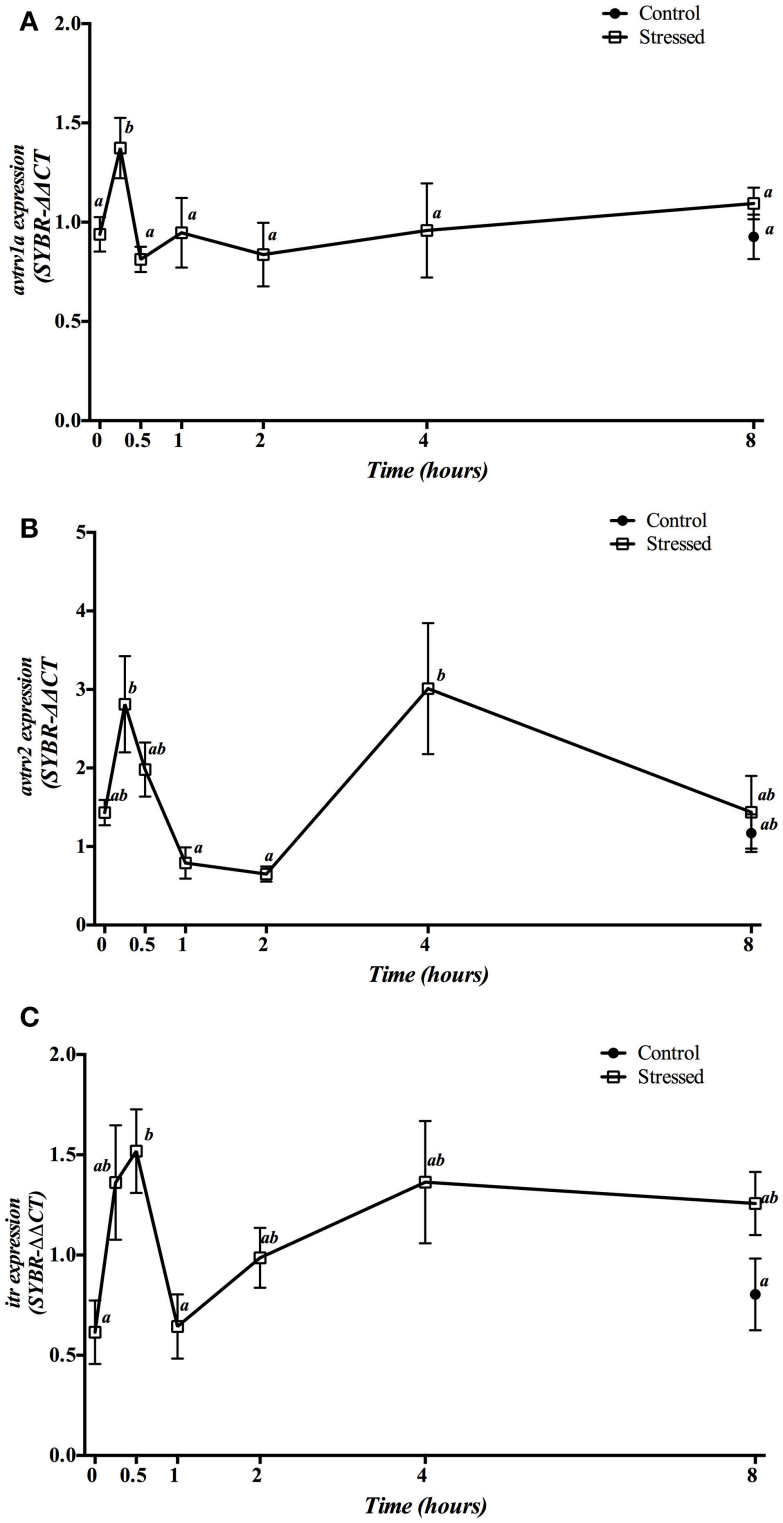
Time course changes in hepatic *avtrv1a* **(A)**, *avtrv2* **(B)** and *itr* **(C)** mRNA expression levels (relative to *actb*) in *S. aurata* exposed to air for 3 min. For further details, see the legend on Figure 1.

Hepatic mRNA expression of *avt* and *it receptors* after 3 min of air exposure is displayed in Figure 8. Expression of *avtrv1a* significantly increased its mRNA levels 15 min post-stress, returning to similar values found in the control group (time 0 h) from 30 min after air exposure onwards (Figure 8A). However, *avtrv2* mRNA expression levels revealed a two-phase pattern with a very pronounced decrease at 1 and 2 h after air exposure (as compared to the levels at 15 min), and a further increase at 4 h after the stress was initiated (Figure 8B). Finally, *itr* gene expression showed a significant increase of its mRNA levels at 30 min followed by a return to the basal levels 1 h post-stress without recovering the control levels after that (Figure 8C). Moreover, no differences on the mRNA expression levels of the three receptors studied were observed between both groups sampled at 8 h post-stress as well as when comparing both control groups (times 0 and 8 h).

## Discussion

To our knowledge, there are no studies that examined the role of Avt/It systems during acute stress events, their presumable interaction with other endocrine axes (hypothalamic-sympathetic-chromaffin cells—HSC, and hypothalamic-pituitary-internal—HPI), and their possible interactions with circadian rhythms. In addition, little is known about the HSC activation after acute stress in *S. aurata*. For this reason, the present study aimed to unravel the activation of different endocrine systems in response to an acute stressor produced by air exposure from the first 15 min until 8 h post-stress.

### HSC and HPI axes

Previous studies in gilthead sea bream indicate that air exposure for 3 min is effective to promote stress system activation (Arends et al., 1999). The stress response involves the recognition of the threat by the central nervous system, with a rapid increase in plasma catecholamines and cortisol values, mediated by both HSC and HPI axes, as a primary response activation. In turn, the activation of these endocrine pathways derivate in plasma and tissue metabolites changes to cope with the energy imposed by the stressor (Rotllant and Tort, 1997; Rotllant et al., 2000; Costas et al., 2011; Sadoul and Vijayan, 2016). Plasma and mRNA expression results obtained in our study about the activation of both HSC and HPI pathways, as well as in the metabolic orchestration, agree with this idea. In fact, the sharp and transitory increase in plasma glucose and lactate perfectly matches with a significant enhancement of both catecholamines at 15 min post-stress, as well as with several players of the HPI axis (see below). Although in our experimental approach we do not have data from the first few minutes post-stress, studies in rainbow trout under acute handling stress showed a very fast increase (in the first 2 min) in plasma catecholamine levels. This is the consequence of the massive release of the hormones from chromaffin cells of the head kidney (Gesto et al., 2013). In addition, our results concerning tyrosine hydroxylase (Th), the rate-limiting enzyme of catecholamine biosynthesis in the head kidney, showed that *th* expression was enhanced during the first hour post-stress and declined slowly after this time, at the same time than plasma catecholamines levels recovered basal values (Figures 3A–C). This suggests that synthetized catecholamines are accumulated for their rapid release following a new stress episode, as well as that their syntheses are turned off till basal levels when the storage and/or release needs have concluded. On the other hand, the concomitant changes in expression levels of hypothalamic factors (*crh, crhbp* and *trh*), adenohypophyseal hormonal precursors (*pomca* and *pomcb*) and the first enzyme involved in cortisol synthesis (*star*), showed that all the components that play a role in the HPI axis are centrally affected by this acute stress model (air exposition). Thus, the gene expression of these endocrine factors independently orchestrates the final release of cortisol hormone from the first 15 min post-exposure. The high *crh* expression level in the first 30 min post stress demonstrates its role in the adaptive process to stressors (Flik et al., 2006), whereas the dual response of *crhbp* expression reveals that the system needs to adapt both factors to face the acute stress situation. In this regard, it is interesting to remark a sharp down-reulation of *crhbp* expression during the first moment of acute stress, demonstrating the modulatory role of this binding protein to activate HPI axis without the necessity of a *crh* stimulation, as well as a complex trade-off of Acth release between Crh and Crhbp (Huising et al., 2004; Flik et al., 2006; Wunderink et al., 2011), as confirmed by plasma cortisol and catecholamine levels 30 min post-stress. In addition to that, the role of Trh in the stress response can not be ruled out as an up-stream regulator of this complex endocrine network, since this hypothalamic hormone has been proposed and demonstrated to cope with its physiological function not only by increasing thyroid stimulating hormone (*tsh*) expression in fish pituitary (Chatterjee et al., 2001; Han et al., 2004) but also to stimulate proopiomelanocortin (Pomc) synthesis (Gorissen and Flik, 2016). Moreover, it seems that in *S. aurata* Trh can acts as a pleiotropic hormone, being involved in the regulation of the synthesis and/or secretion of several pituitary hormones, or even acting as a neuromodulator in other regions of the brain (Ruiz-Jarabo et al., 2018).

At the pituitary level, two proopiomelanocortin (Pomcs) have been described in several teleost species to participate in the endocrine stress response, including *S. aurata* (Cardoso et al., 2011; Wunderink et al., 2012; Saccol et al., 2018): (i) Pomca, responsible for α-Msh synthesis, and (ii) Pomcb, responsible for Acth production. In our study, both precursos showed different pattern of changes (Figure 2). Enhancement of *pomcb* expression between 30 min and 2 h post stress further confirm an activation of HPI axis with high cortisol synthesis and release. This has also been demonstrated by the cortisol plasma levels found after air exposure (see above), as well as by the *star* gene expression in the head kidney. The last one orchestrates the rate-limiting step in the production of steroids hormones as cortisol, by regulating cholesterol transfer within the mitochondria. However, the decrease observed in *pomca* expression just after the challenge (from 15 and 30 min post-stress) suggests that this Pomc paralogue has not a role during acute stress, as previously hypothesized in this fish species (Toni et al., 2015).

Finally, it is interesting to remark that recent studies pointed out the existence of daily variation in different players involved in the stress response, like plasma cortisol, catechoalmines or glucose mobilization, in the sensitivity of adenohypophyseal Acth cells to Crh, or the interrenal gland to Acth (Le Bras, 1984; López-Olmeda et al., 2013; López-Patiño et al., 2014; Vera et al., 2014), as it was also confirmed by some of our results in the gilthead sea bream. In this sense, little is known on the overall presence of circadian rhythms and time dependent effects after an acute stress event, although the influence of the time of the day in the stress response seems to be species dependent (Cowan et al., 2017). Therefore, the stress response described in fish species must be always referred respect to the time of the day where the stressor was applied, and therefore to the geographical position and time of the year.

### Avt/It systems

In addition to the clasical point of view of HSC and HPI axes as the main endocrine cascades to face the stress response caused after a large variety of challenges, the role of Avt in the activation of teleostean stress system at central level has been proposed in several studies (Kulczykowska, 2001; Balment et al., 2006; Mancera et al., 2018). This role was based on the finding than hypothalamic neurons responsible for the synthesis and release of this neuropeptide innervate adenohypophyseal cells (Moons et al., 1989). Furthermore, Avt and Crh sinergize to stimulate Acth release in trout pituitary *in vitro*, suggesting a role of Avt in the central regulation of the HPI axis. Moreover, previous studies in *S. aurata* highligthed a possible role of vasotocinergic and isotocinergic systems in chronic stress situations produced by high stocking densities (Mancera et al., 2008; Skrzynska et al., 2018), hyper- and hypo-osmotic transfers (Martos-Sitcha et al., 2013, 2014a), cortisol treatment (Kalamarz-Kubiak et al., 2014; Cádiz et al., 2015), or food-deprivation and refeeding (Skrzynska et al., 2017). In our present study, gene expression profiling of both hypothalamic Avt and It precursors (*avt* and *it*, respectively) and their specific receptors (*avtrv1, avtrv2*, or *itr*) were assessed in control and air-exposed sea breams. As a remarkable result of this experiment, *avt* and *it* precursors showed a similar pattern of changes with a sharp increase at 1 h post-stress, suggesting a stimulatory role of vasotocinergic and isotocinergic systems during the first moment of stress. As a consequence, Avt and It released could be used to stimulate several metabolic pathways to produce fuel that can be used within the central nervious system for the synthesis of neurotransmitters or for other neuronal or glial cell activities (Soengas and Aldegunde, 2002; Sangiao-Alvarellos et al., 2004, 2006). These results are also in line with the gene expression of Avt and It receptors. In this regard, our results make us to hypothesize that *avtrv1a* may be involved in the control of the endocrine system during the first moments of stress, whereas *avtrv2* can control different metabolic enzymes for extra energy supply and repartitioning process after that to cope with the physiological action required. This interesting feature, where different putative roles of both Avt receptor genes could orchestrate several physiologycal functions, has been previously demonstrated in *S. aurata* under environmental salinity challenges (Martos-Sitcha et al., 2014a), or even after an intraperitoneal administration of cortisol (Cádiz et al., 2015). Meanwhile, the expression of *avtrv1a* at hypophyseal level can be explained by: (i) an inhibitory effect of cortisol during the first minutes post-stress to finely mediate feedback mechanisms to maintain homeostasis of the HPI (Arends et al., 1999), and (ii) the ulterior stimulation of Avt release when cortisol levels start to return to their basal levels (Martos-Sitcha et al., 2013). This fact is also concomitant and complemented by *avtrv2*, which is predominantly expressed in the pituitary of unstressed fish (Martos-Sitcha et al., 2014a), and can be affected by circadian variations at central level (Lema et al., 2010; present results) as other components of the vasotocinergic system (Rodríguez-Illamola et al., 2011). However, to our knowledge, this is the first study showing the important role of Avt receptors during the first moments of the acute stress response.

The physiological functions of the isotocinergic system in teleosts are not well-established, although some authors suggest a role of It hormone in the stress system, enhancing its plasmatic levels under stress situation such as dominance and subordination in *Oreochromis mossambicus* (Almeida et al., 2012), or changes in the reproductive and social status in *Gasterosteus aculeatus* (Kleszczynska et al., 2012). In *S. aurata*, treatment with slow-release implants containing cortisol or different chronic stress situations (i.e., high stocking density and fasting conditions) induce changes in both central (brain and pituitary) as well as peripheral (liver and head kidney; see below) players of the isotocinergic system. In addition, it is interesting to note that a diurnal variation exists in the hypothalamic *itr* gene expression, which agrees with previous studies in the Amargosa pupfish (*Cyprinodon nevadensis amargosae*) (Lema et al., 2010), although its physiological significance remains unclear. Unfortunately, it was not possible to measure plasma Avt and It levels due to the small size of the animals. This could have provided, together with catecholamines and cortisol, a more complete view about the role of these nonapeptides in metabolic and stress pathways. Nonetheless, the study of their specific recceptors in important target tissues also give us valuable information about these processes.

The head kidney is a complex tissue of particular importance due to its mixed cellular diversity and multiple functions associated with them (Fierro-Castro et al., 2015). The endocrine cascade after acute stress will activate catecholamines synthesis in the chromaffin cells and cortisol synthesis in the steroidogenic cells of the interrenal tissue (Baker et al., 1996; Wendelaar Bonga, 1997), as suggested from our results of *th* and *star* expression at head kidney, as well as the circulating levels of these hormones (see above). In addition, changes in both *avtr*s (*v1a* and *v2* types) and *itr* gene expression make us to hypothesize that Avt and It nonapeptides participate in feedback mechanisms directly in the head kidney regulating the synthesis/release of cathecholamines and/or cortisol hormones, where *avtrv2* is also able to help in the modulation of the diurnal variation of the HPI observed (see above). In fact, a relationship between cortisol (and presumably catecholamines) and both vasotocinergic and isotocinergic systems has been proposed for *S. aurata* following both *in vivo* (Cádiz et al., 2015) and *in vitro* (Kalamarz-Kubiak et al., 2014) studies. In this species, it has been also suggested that chronic stress triggered by high density or food deprivation (Mancera et al., 2008; Skrzynska et al., 2017) boost the endocrine function of Avt and It pathways to orchestrate the energy requirement against the new physiological status. Moreover, our study of acute stress indicates that plasma cortisol mirrors the gene expression levels of *star*. It has been found in previous studies than this protein mediates a rapid activation of cortisol production from mammals to fish (Liu et al., 1996; Ariyoshi et al., 1998; Hagen et al., 2006). This is done by a regulation mediated by cAMP-dependent protein kinase system and protein kinase C-dependent processes (Nishikawa et al., 1996), among others, which interestingly control the signaling pathways of Avt and It receptors (Hausmann et al., 1995; Wargent et al., 1999; Warne, 2001). Nonetheless, the effect of cortisol and catecholamines, together with feedback connections between these two hormonal systems, and the differential effects on shared target cells, make a complex interpretation of these relationships (Gerwick et al., 1999). However, further studies related with the specific isolation of different cell types (chromaphin cells and steroidogenic cells) and the evaluation of *avtr'*s and *itr* in each of them will be neccesary in order to establish the specific role of Avt/It during acute or chronic stress in this important stress-related tissue.

After a stressfull situation, the stimulation and regulation of several pathways of the hepatic metabolism are needed to activate complementary nechanisms and to trade-off the effects of high levels of catecholamines and cortisol observed, with the subsequent energy release into the internal medium of the animal (Mommsen et al., 1999; Laiz-Carrión et al., 2002, 2003). Avt and It receptors also share intracellular signaling pathways with different metabolic enzymes, regulating their activities to control the energy production and supply. In this regard, the incorporation of inorganic phosphate in phosphatidyl inositol routes, which acts as a substrate for phospholipase C, as well as the increase of Ca^2+^ to activate hepatic phosphorilase, can be considered as important players in the metabolic response observed and mediated by these receptors (Kirk et al., 1977, 1979; Alemany et al., 1981). This has previously been suggested in *S. aurata* after other stress challenges (Martos-Sitcha et al., 2014a; Cádiz et al., 2015; Skrzynska et al., 2017). For instance, the increase observed in the gene expression of the three receptors (*avtrv1a, avtrv2*, and *itr*) analyzed in liver, together with the changes observed in plasma and tissue metabolite levels, suggest that these receptors are associated with the primary adaptive response, related with the enery metabolism, during the first moments after the challenge, whith a putative function of *avtrv2* and *itr* also several hours post-stress.

## Summary and future perspectives

This study shows that acute stress situations, like the one produced by air exposure during handling procedures in aquaculture practices, stimulates both HPI and HSC axes in *S. aurata*, activating the transcriptomic response of different endocrine factors (*crh, crhbp, trh, pomc*s) at hypothalamic and hypophyseal levels, as well as the *star* and *th* expression in the head kidney. Furthermore, a transitory enhancement of the main stress hormones (catecholamines and cortisol) ocurrs and could be involved in liver metabolism stimulation. Changes in *avt* and *it* gene expression, as well as in their specific receptors (*avtrv1, avtrv2*, and *itr*) at central (hypothalamus and pituitary) and peripheral (liver and head-kidney) locations, also showed that vasotocinergic and isotocinergic systems could have a role in several physiological changes induced by air exposure, including metabolic and energy repartitioning proceses as well as the control of synthesis and release of several hormones as the final product of different endocrine pathways, as suggested by the tissues analyzed and the related-functions involved in acute stress events. As a future possibility we think it would be interesting to check changes of the different endocrine players assessed here (HPI, HSC, vasotocinergic, and isotocinergic systems) in shorter time after acute stress, as well as to characterize the specific co-localization of Avt and It receptors between the mixed cellular diversity of the head kidney. Altogether, our results demonstrate the cooperation among different endocrime pathways, and suport the use of these parameters as putative biomarkers to obtain reliable information about the homeostatic load of fish to improve the rearing and handling conditions of this high commercial value species.

## Author contributions

JMa and JM-S: conceived and designed the study; AS, EM, MB, and JM-S: carried out experimental procedures; AS, EM, MB, FN, JMí, GM-R, JMa, and JM-S: analyzed and interpreted the data; AS, JMa, and JM-S: wrote the original draft; All authors reviewed, edited and approved the final manuscript.

### Conflict of interest statement

The authors declare that the research was conducted in the absence of any commercial or financial relationships that could be construed as a potential conflict of interest.
